# Splenic Torsion From a Wandering Spleen

**DOI:** 10.7759/cureus.69369

**Published:** 2024-09-13

**Authors:** Rachel M Hernandez, Benjamin J Duddy, Kyle J Iverson

**Affiliations:** 1 General Surgery, Keesler Medical Center, Biloxi, USA

**Keywords:** abdominal radiology, spleen, splenectomy, splenic torsion, wandering spleen

## Abstract

Wandering spleen is a unique condition defined as a hypermobile spleen with a sole attachment to its vascular pedicle. The most common complication is torsion of the spleen, resulting in a surgical emergency. Early diagnosis and prevention of splenic infarction are the mainstays of treatment to help preserve immunologic function. We report a case of torsion of the wandering spleen in a 23-year-old active-duty male with a prior childhood history of duodenal atresia. His abdominal examination demonstrated a palpable midline mass, which was confirmed by CT abdomen and pelvis to be a wandering spleen with twisting of the vascular pedicle. The patient was taken for an exploratory laparotomy, where an infarcted spleen was identified with associated twisting of the splenic hilum, and a splenectomy was performed. Post-operatively, the patient is healthy and symptom-free at the three-month follow-up and has returned to active duty.

## Introduction

Wandering spleen is when the spleen is in a location other than its normal anatomic location in the left upper quadrant. The spleen is usually anchored by four ligaments: gastrosplenic, splenorenal, splenocolic, and splenophrenic [1,2]. If there is failure of development or trauma to any of the ligaments, this predisposes the spleen to wander. The main concern with the change in laxity of the anchoring ligaments is that this creates an elongated vascular splenic pedicle [3]. The consequence of this is an increased likelihood of torsion and subsequent vascular compromise of the spleen [4]. The current management of splenic torsion is based on whether or not the vasculature has been compromised. We present a case of wandering spleen resulting in splenic torsion in a patient with a childhood history of duodenal atresia.

## Case presentation

A 23-year-old male active US military service member with a reported history of duodenal atresia requiring surgical intervention during infancy presented to the emergency department for abdominal pain and intractable nausea. Of note, two days prior to this visit, the patient was discharged from the hospital after the resolution of his acute pancreatitis. During his index hospitalization, he had a CT of the abdomen and pelvis, demonstrating swirling of the mesentery with splenomegaly and mention of the spleen in the right lower quadrant. He subsequently underwent a right upper quadrant ultrasound, which showed marked splenomegaly with an ectopically positioned spleen concerning significant malrotation, with the spleen appearing within the right midline upper abdomen extending to the lower midline abdomen. Small collaterals near the splenic hilum were visualized. Initially, his abdominal pain was attributed to his pancreatitis, and no surgical consultation was placed. He underwent conservative management for his pancreatitis. On hospital day 2, his abdominal pain resolved, his diet was advanced, and he was discharged.

The patient reported that after discharge, he was doing well until that evening of presentation to the emergency department when he started experiencing lightheadedness and abdominal pain after consuming a light meal. The patient’s blood pressure was 132/76 mmHg, heart rate was 80 bpm, respiratory rate was 18 bpm, afebrile, and saturating at 98% on room air. At the time of admission, the patient’s CBC was within normal limits, except that his platelet count was unquantifiable, with a laboratory addendum stating massive platelet clumping (3+) and normal adequacy (Table 1). Basic metabolic panel (BMP) was within normal limits except for borderline hypokalemia (Table 1).

**Table 1:** Laboratory analyses at time of admission and post operatively.

Parameters	Admission labs	Post-op day 1 labs	Reference values
WBC (× 10^3^/μl)	4.84	12.6	4.5–11.0
Hemoglobin (g/dl)	12.6	11.8	12.0–15.0
Hematocrit (%)	37.9	35.3	36.0–48.0
Platelets (× 10^3^/μl)	Unquantifiable	184.0	150.0–450.0
Sodium (mmol/l)	135.0	137.0	136.0–145.0
Potassium (mmol/l)	3.5	3.9	3.5–5.1
Chloride (mmol/l)	100.0	103.0	98.0–107.0
CO_2_ (mmol/l)	30.0	29.0	21.0–32.0
BUN (mg/dl)	17.0	14.0	7.0–18.0
Creatinine (mg/dl)	1.1	1.0	0.6–1.3
Glucose (mg/dl)	113.0	100.0	74.0–106.0

**Figure 1:**
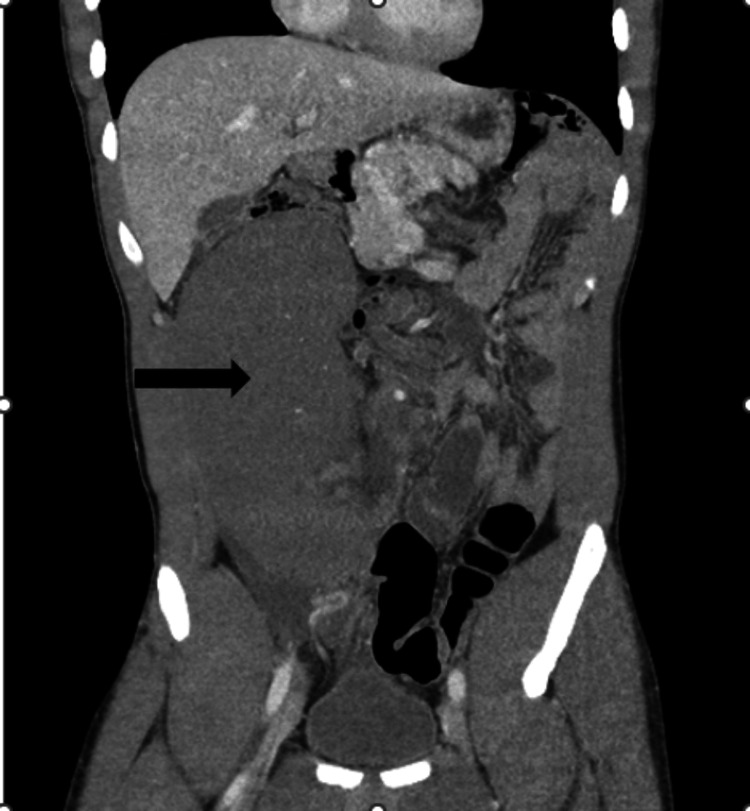
CT abdomen and pelvis, coronal view, slice 27. Black arrow representing wandering spleen.

A CT of the abdomen and pelvis was obtained, redemonstrating the swirling and twisting of the mesentery along the superior mesenteric artery in the mid-abdomen. The report mentioned a large, obstructed bowel loop versus a cystic lesion identified in the right mid-abdomen measuring 20 cm × 10 cm × 10 cm (Figure 1).

**Figure 2:**
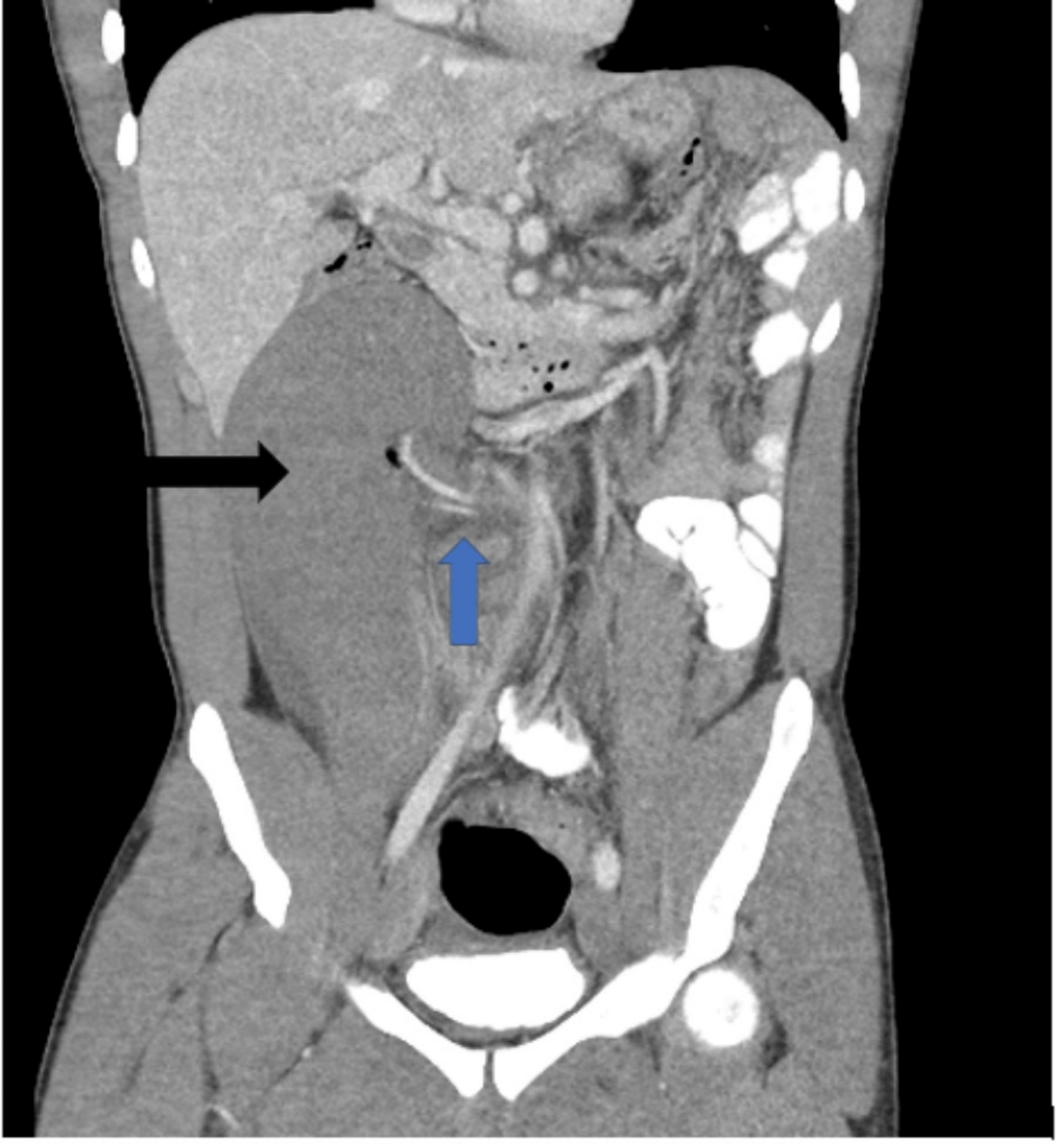
CT abdomen and pelvis, coronal view, slice 34. Black arrow representing wandering spleen. Blue arrow demonstrating splenic pedicle.

The patient ultimately underwent a repeat CT abdomen and pelvis with oral contrast. The radiology report described a wandering spleen within the right mid and lower abdomen, which was enlarged and hypoenhancing (Figure 2).

**Figure 3:**
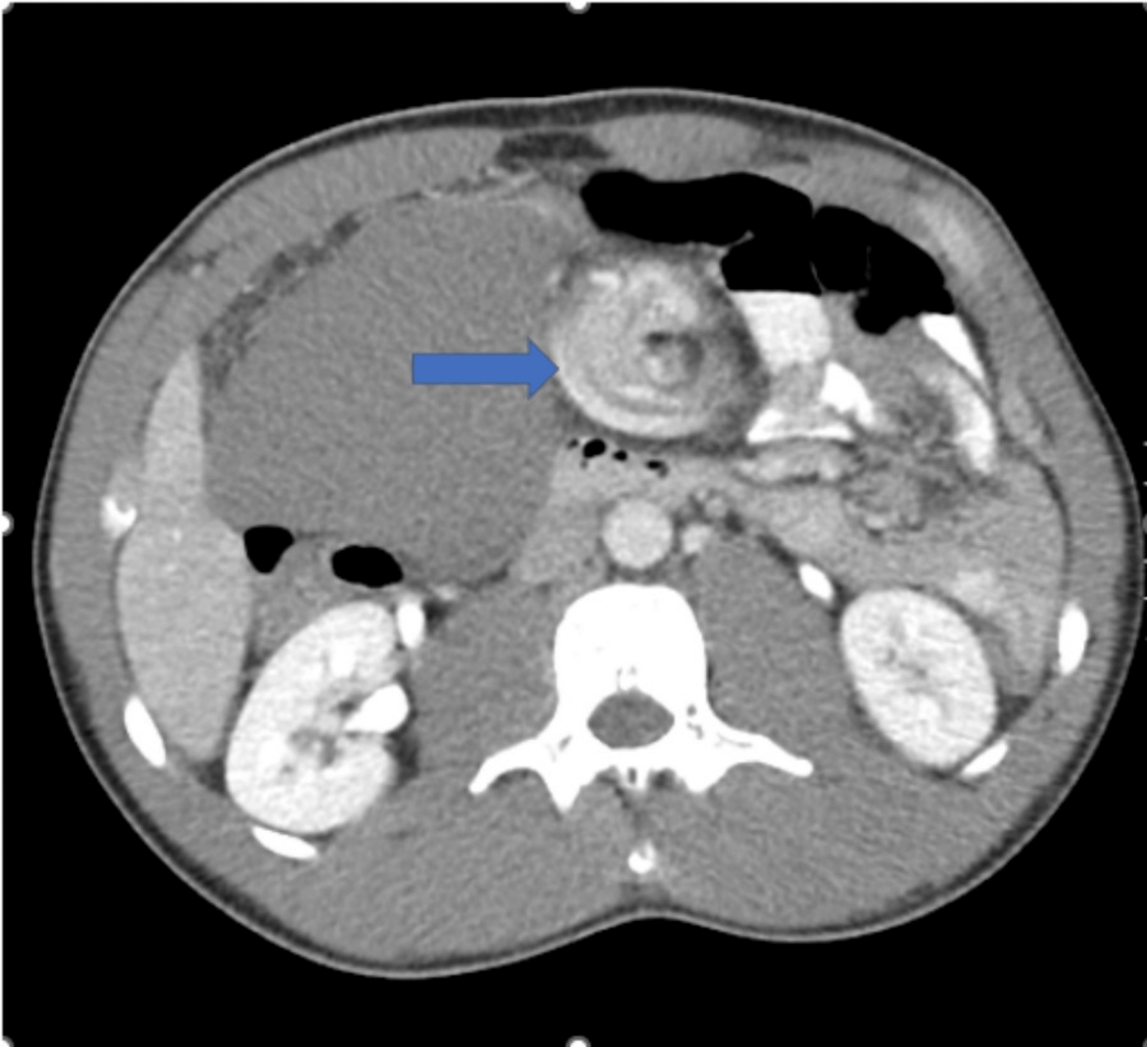
CT abdomen and pelvis, axial view, slice 36. Blue arrow representing twisting of the splenic pedicle.

There was an associated twisting of the vascular pedicle, resulting in splenic vein occlusion (Figure 3).

After a thorough discussion, the patient was taken to the operating room for an exploratory laparotomy with splenectomy. The patient was American Society of Anesthesiologists (ASA) class I.

**Figure 4:**
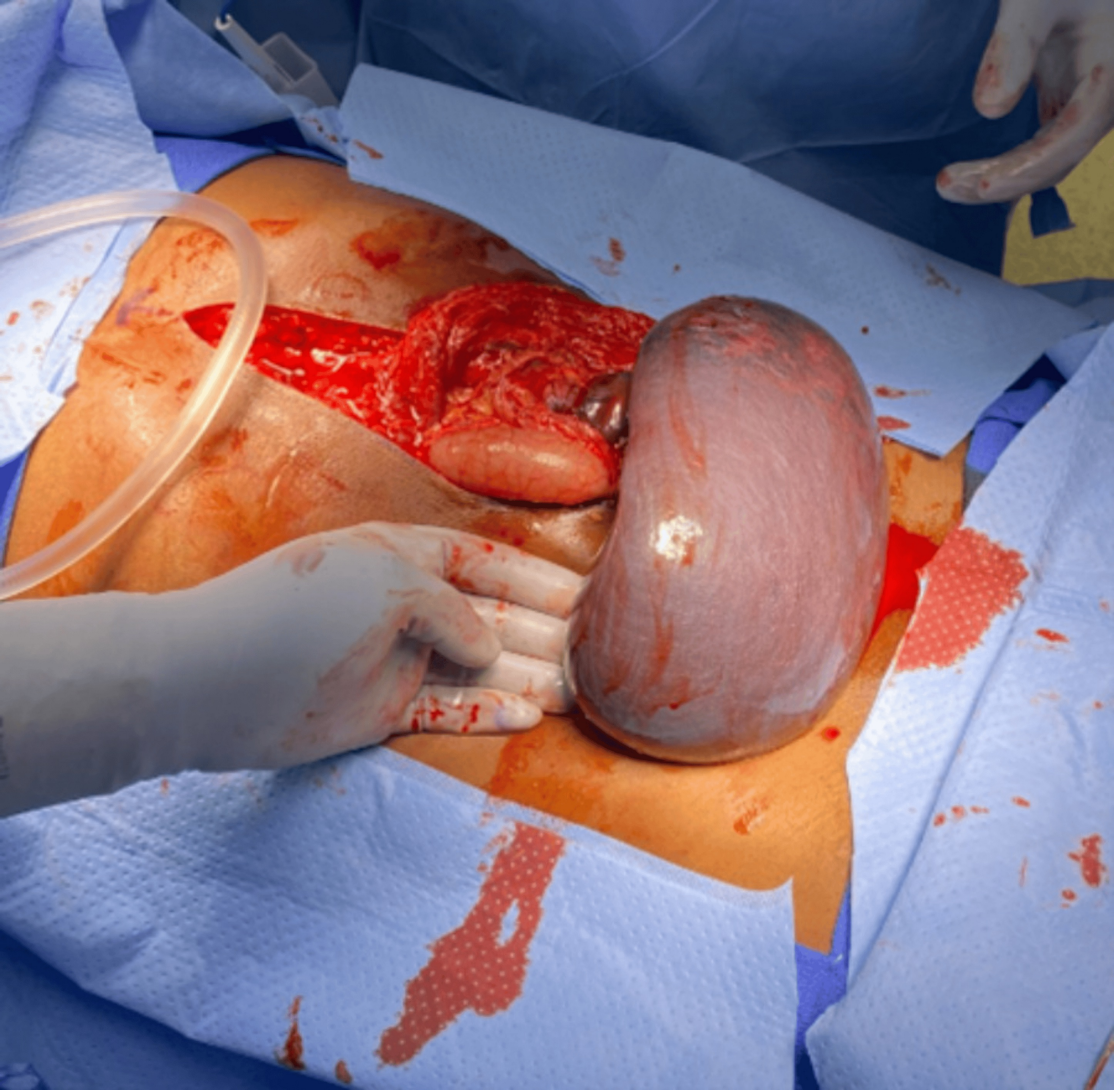
Presentation of the spleen from the abdomen after detorsion.

Upon entrance into the abdomen, the spleen was midline with obvious splenomegaly and was dusky in appearance. The spleen was delivered from the abdominal cavity, and torsion of the hilum was noted (Figure 4).

**Figure 5:**
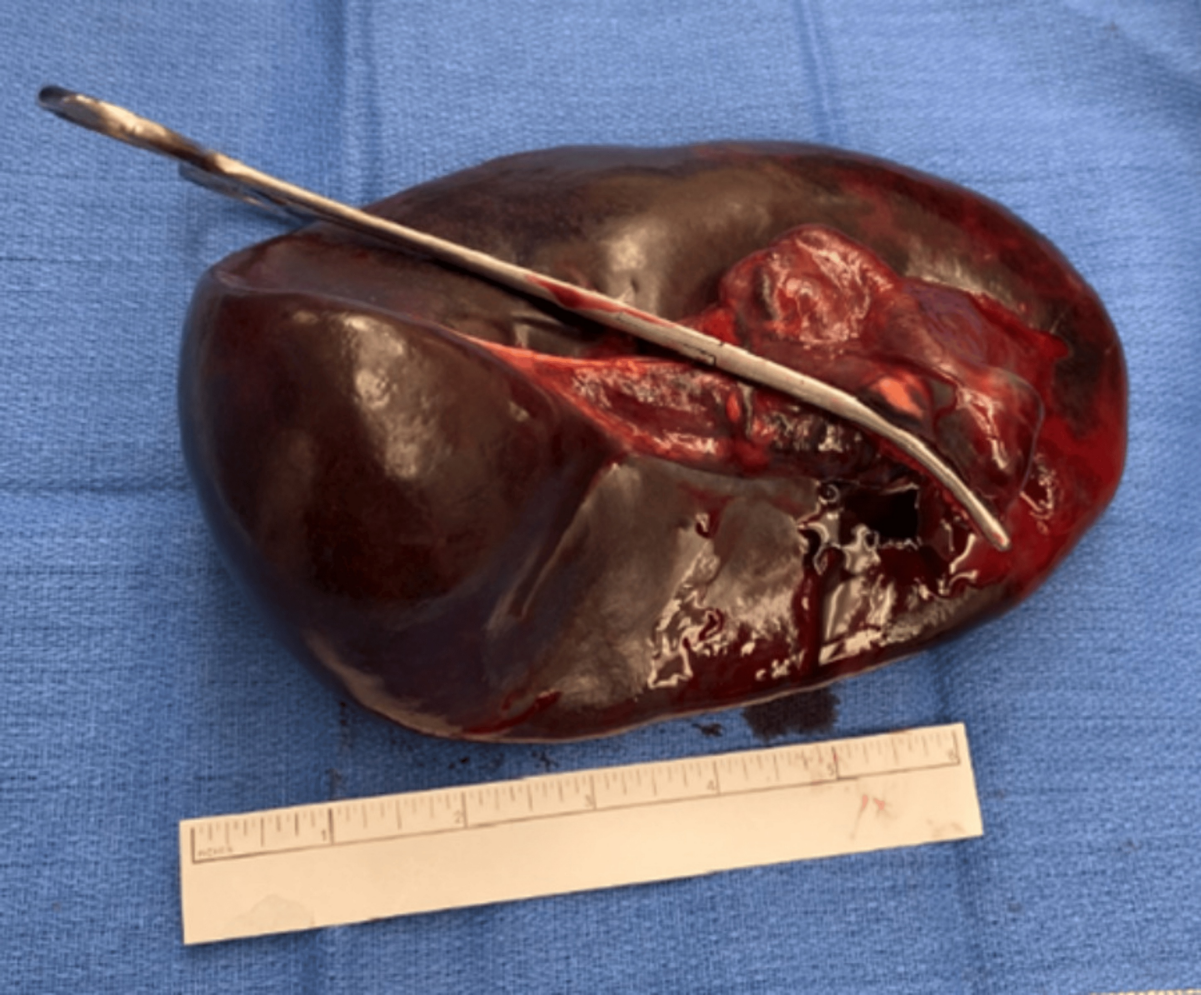
Spleen with Kelly clamp overlying the ligated splenic pedicle.

The omentum was wrapped around the hilum and splenic vessels as well. After untwisting the omentum, the pedicle was transected close to the hilum, and the spleen was released via suture ligation and electrocautery (Figure 5).

The small bowel was run from the terminal ileum to the ligament of Trietz without any significant findings, and the abdomen was closed.

On postoperative day 1, the patient’s pain was well controlled, and he was started on a clear liquid diet. He had a reactive leukocytosis, as expected, secondary to surgery and spleen removal (Table 1). BMP was unremarkable in terms of the resolution of the patient’s hypokalemia and improved platelet count (Table 1). The patient continued to progress during his admission, with advancement to a regular diet on postoperative day 2 and return of bowel function on postoperative day 3. He was successfully discharged home and followed up in the clinic two weeks later, where he received his post-splenectomy vaccines. The pathology report revealed a benign spleen with venous thrombosis and infarctive changes.

## Discussion

Wandering spleen, also known as hypermobile spleen, is a failure to develop the normal splenic attachments. The spleen is normally fixed in the left upper quadrant by the gastrosplenic, splenorenal, splenocolic, and splenophrenic ligaments. A failure of these attachments results in abnormal splenic anatomy. The spleen originates from mesenchymal cells in the dorsal mesogastrium, which later develops into the greater omentum [1]. During embryologic development, the spleen migrates to the left upper quadrant. Failure of fusion of the dorsal mesogastrium produces an abnormally long vascular pedicle, as the splenic artery and tail of the pancreas reside in the splenorenal ligament [2]. 

The etiology of the ligamentous laxity can be congenital, as stated above, or acquired. Congenital anomalies can alter the normal anatomic position of the spleen, leading to a higher likelihood of a wandering spleen. There have been case reports discussing the association of wandering spleen with gastric volvulus. However, no reports have discussed the association with duodenal atresia [3]. While our patient’s past medical history is unique, the inciting factor for his wandering spleen is more likely attributed to his surgical intervention in infancy (i.e., acquired), rather than a failure in intestinal recanalization seen in duodenal atresia [4].

The acquired form often results from a weakening of the ligaments commonly seen during pregnancy secondary to elevated hormonal levels, trauma, or surgical manipulation [5]. Regardless of the etiology, congenital or acquired, the abnormally long pedicle creates the possibility of torsion. As a result, the spleen is then susceptible to either a partial or complete infarction.

The clinical presentation varies from chronic, intermittent abdominal pain to an acute abdomen. The former is often secondary to the spleen undergoing a partial torsion and then subsequent detorsion [5]. On clinical examination, one can appreciate a firm, moveable abdominal mass. However, the diagnosis of a wandering spleen is not often made based on clinical diagnosis alone. While plain films and barium-based studies can appreciate the superior-medial displacement with extrinsic compression of the spleen on the surrounding structures, these findings are often non-specific and require further imaging [6].

Computed tomography is the preferred imaging modality. The most common findings on CT for a wandering spleen and splenic torsion include the absence of the spleen in the left upper quadrant, a lower abdominal or pelvic mass with attenuation changes different from normal splenic parenchyma, a whorled appearance of the splenic vessels, and possible necrosis of the distal pancreatic tail [6,7]. Of the possible CT findings, the most specific is the whorled appearance of the splenic vessels around the splenic hilum.

The management of wandering spleen and splenic torsion relies on the presence of compromised vasculature. If there is evidence of infarction, then splenectomy is still the treatment of choice. If vascular inflow and outflow are preserved, some authors propose splenopexy, if possible, to help preserve the immunologic functions. Bouhaddouti et al. repeated the Stringel technique and demonstrated stabilization of the spleen in the left upper quadrant utilizing two 3-0 silk sutures running from the upper and lower ends of the hilum to the posterior peritoneum [8]. This was able to provide enough support to prevent further torsion [8,9].

## Conclusions

A wandering spleen is a unique anatomical anomaly that has multiple etiologies. Regardless of how a wandering spleen develops, the most important consideration in management is the viability of the splenic pedicle. Despite being rare, a torsion of a wandering spleen can result in a serious abdominal emergency. Prompt diagnosis can help prevent splenic infarction and ultimately lead to splenic preservation. In instances where splenic torsion has led to substantial infarction or thrombosis, splenectomy is still the preferred management. Otherwise, splenopexy can be considered.
